# Influence of Microfibrillated Cellulose Additive on Strength, Elastic Modulus, Heat Release, and Shrinkage of Mortar and Concrete

**DOI:** 10.3390/ma14226933

**Published:** 2021-11-16

**Authors:** Yurii Barabanshchikov, Hien Pham, Kseniia Usanova

**Affiliations:** Institute of Civil Engineering, Peter the Great St. Petersburg Polytechnic University, 195251 St. Petersburg, Russia; ugb@mail.ru (Y.B.); phamthehien710@gmail.com (H.P.)

**Keywords:** concrete, mortar, nano/microfiber cellulose, testing, strengths, tensile modulus, shrinkage, heat release

## Abstract

This work aimed to study the effect of a microfibrillated cellulose additive on strength, elastic modulus, heat release, and shrinkage of mortar and concrete. The dosage of the additive varies from 0.4 to 4.5% by weight of the cement. The change in strength with an increase in the dosage of the additive occurred in a wave-like manner. The uneven character of the change in the results also took place in the determination of heat release and shrinkage. In general, heat release and shrinkage decreased at increasing additive dosage. The additive showed the greatest decrease in the heat release of concrete at a content of 2%. The heat release of concrete practically differed little from the exotherm of the standard at an additive content of 1 and 1.5%. The addition of microfibrillated cellulose additive in small (0.5%) and large (1.5%) amounts reduced shrinkage compared to the reference, and at an intermediate content (1%), the shrinkage was higher than in the reference specimens. In this case, the water evaporation rate from concrete increased with an increase in the additive. With an increase in the additive dosage, the modulus of elasticity decreases. Thus, the microfibrillated cellulose additive provides concrete with lower values of the modulus of elasticity, heat release, and shrinkage, and the additive is recommended for use in concretes with increased crack resistance during the hardening period. The recommended additive content is 0.5% by weight of cement. At the specified dosage, it is possible to provide the class of concrete in terms of compressive strength C35/45.

## 1. Introduction

Much attention is paid to a direction in concrete technology associated with using nanostructures of carbon, oxides of silicon, titanium, iron, and others [1,2,3,4,5]. Among the modifying additives, the use of microfibrillar cellulose in cement composites is being actively studied. A biopolymer additive to concrete made from beet pulp (SBP), a waste product from the sugar industry, has appeared on the building materials market. 

There is evidence [6] that SBP cellulose can have great potential for a series of materials in which rheology is essential. Unlike most celluloses derived from secondary wall fibers, SBP cellulose is a typical primary wall cellulose called parenchymal cell cellulose [7]. The inclusion of natural fibers in the structure of the cement composite is a means of minimizing the problem of cracking, increases the impact toughness, flexural strength, and changes the nature of fracture of brittle materials towards more ductile cracking resistance [8]. Plant fibers have additional advantages, such as abundance, renewability, low density, and high mechanical strength [9]. However, it has been reported that the chemical components and soluble sugars contained in plant fibers slow down the hydration of cement-based mixtures [10,11]. This slow-up was observed when testing fibers from sugarcane pulp [12], bamboo flakes and oil palm leaves [13], hemp [14], and tropical wood [15]. Fiber pectin can form complex molecules with calcium ions and may be responsible for the observed set retardation [14].

It was established [16] that pore water with severe alkalinity damages macromolecular chains, due to hydrolysis of cellulose, which causes their rupture, and, as a consequence, a decrease in the degree of polymerization of cellulose chains. To evaluate the degradation of composites in [17], an accelerated aging process was carried out using 200 moistening and drying cycles. In hybrid composites, nanofibrillated cellulose has improved mechanical properties compared to a composite without nanofibers.

The investigated additive Pro-Flowstab is a ready-to-use aqueous suspension of microfibrillated cellulose from beet pulp, with a pH of about four and a density of about 1 g/cm^3^. Regulating the mixture’s rheological characteristics is carried out by changing the additive’s dosage, which, according to the manufacturer’s recommendation, varies from 0.5 to 2.5% of the cement mass and is introduced into the concrete mixture after adding water.

According to the manufacturer’s data, microfibrillar cellulose Pro-Flowstab, obtained from the primary wall of parenchymal cells, consists of microfibrils, including elementary nanofibrils with a 2–4 nm diameter, consisting of 18–24 strands of the cellulose polymer. According to the data of [18], the Pro-Flowstab additive consists of nanofibrillated cellulose, which is a long bundle of fibers with a diameter of about 2 nm, with an admixture of larger microfibrillar structures with an average transverse size of about 100 nm. Such materials are heterogeneous and contain fibers, fiber fragments, microfibrils, and nanofibrils. This material is called microfibrillated cellulose (MFC) [19]. In addition to Pro-Flowstab, an additive based on microcellulose fibers from sugar beet pulp is known under the trade name Betafib® MCF, which does not contain lignin. The additive material comprises at least 60% cellulose, 0.5–10% pectin, and 1–15% hemicellulose and has a transverse particle size in the range of 25–75 microns. When added to liquid compositions, the additive leads to an increase in viscosity [20].

There is no generally accepted classification of supramolecular structures in the literature. The terms used for nano- and micro-sized cellulosic materials are not fully established. The same material may have different names, or the same terms may be used for different materials. M. Ioelovich gives the following characteristics of the micro- and nanostructures of fibrillated cellulose. The units of cellulose macromolecules connected by hydrogen bonds of hydroxyl groups form the primary elements of the supramolecular structure, elementary nanofibrils with transverse dimensions of 3–10 nm, depending on the origin of cellulose. Due to the high specific surface area, primary nanofibrils tend to aggregate to form fibrillar bundles called microfibrils. In turn, elementary nanofibrils and their beams contain crystallites and amorphous domains.

The authors of [21] consider microfibrillated cellulose (MFC) fibers with transverse dimensions in the range of 10–40 nm, consisting of aggregates of cellulose microfibrils (Microfibril), and a limited number of nanofibers 3–10 nm. Microfibrils have a minor diameter of 2–10 nm and a considerable length, more than 10 microns, due to which they are prone to aggregation, which adversely affects the mechanical properties of cement composites, as it creates a weak bond at the contact between the fibers and cement hydrates [22].

The authors of [23,24] distinguish two types of nanocellulose: nanocrystals and nanofibrils. The former is obtained by treating the fibers with acids, and the latter by mechanical destruction. Based on the above, Pro-Flowstab should be classified as MFC, as the exact ratio and morphology of microcellulose and nanocellulose are unknown. Comparison of the effect of these cellulose structures on the viscous fracture of reactive powder concrete showed [25] that with the same additive content (3% by weight of cement), specimens with microcellulose had tensile fracture energy upon splitting that was three times higher than specimens with nanocellulose. Microcellulose also provided higher energy absorption during the three-point bending of the specimens. At the same time, in contrast to microcellulose, nanocellulose with increasing dosage did not require an increase in water-cement ratio and superplasticizer.

At an early age (in the first 12 h), the addition of cellulose nanofiber reduces cement hydration, and then nanofibrils accelerate cement hydration. Furthermore, the hydration peaks increase with increasing nanofiber content. So, the general tendency is that cement hydration is enhanced by nanofibrils [26]. A notable feature of cellulose nanofibers is their hydrophilicity [27], ensuring their adhesive interaction with cement particles in the concrete mixture.

The variety of structures and morphology of cellulose fibers does not allow transferring the test results of some materials to others. In each case, studies should be carried out on specific types of cellulose fibers. The Pro-Flowstab additive is poorly understood. Only its rheological properties and strength in the composition of cement stone are known [18]. Therefore, the purpose of this work is to study the physical and mechanical properties, such as strength, modulus of elasticity, heat release, and shrinkage, of concrete with the microfibrillated cellulose additive.

## 2. Materials and Methods

### 2.1. Materials

This study uses the following materials.

1. Portland cement without mineral additives CEM I 42.5 N produced by JSC "Mikhailovcement" (Ryazan Oblast, Russia), according to Russian State Standard GOST 31108-2016 “Common cements. Specifications” [28], with a specific surface of 325 m^2^/kg, a normal density of 25.1%, mineralogical composition: C_3_S = 61.1; C_2_S = 17.8; C_3_A = 5.7; C_4_AF = 13.4%.

2. Grade 1 sand for construction works from the Sestrinsky open-pit mine, according to Russian State Standard GOST 8736-2014 “Sand for construction works. Specifications” [29] with a fineness modulus of 2.5, fraction 0–5 mm, with clay content and dust particles no more than 1.5%.

3. Granite crushed stone, fraction 5–10 mm, "LSR-Basic Materials North-West" (Leningrad region, Russia) according to Russian State Standard GOST 8267-93 “Crushed stone and gravel of solid rocks for construction works. Specifications” [30], the grade for crushing: 1200.

4. Superplasticizer: MC-Techniflow 178 (hereinafter SP) manufactured by MC-Bauchemie (St. Petersburg, Russia).

5. Additive Pro-Flowstab based on nano/microfibrillated cellulose from beet pulp in the form of an aqueous suspension, with a density of about 1 g/cm^3^. The additive produced by “Bang & Bonsomer Group Oy” (Helsinki, Finland).

### 2.2. Methods

The experiments were carried out to evaluate the Pro-Flowstab additive to increase the ultimate tensile deformation of concrete, which is included in the formula for the criterion of concrete crack resistance. The dosage of the additive in the experiments with the mortar was from 0.4% to 4.5% by weight of cement, and it was 1%, 1.5%, and 2% in the experiments with concrete. In the first case, it was decided to expand the additive dosage limits in comparison with the manufacturer’s recommendations (0.5–2.5%). In the second case, the additive content was in accordance with the recommendations. The primary test in this work was to determine the elastic modulus. 

#### 2.2.1. Mortar Testing

**Figure 1:**
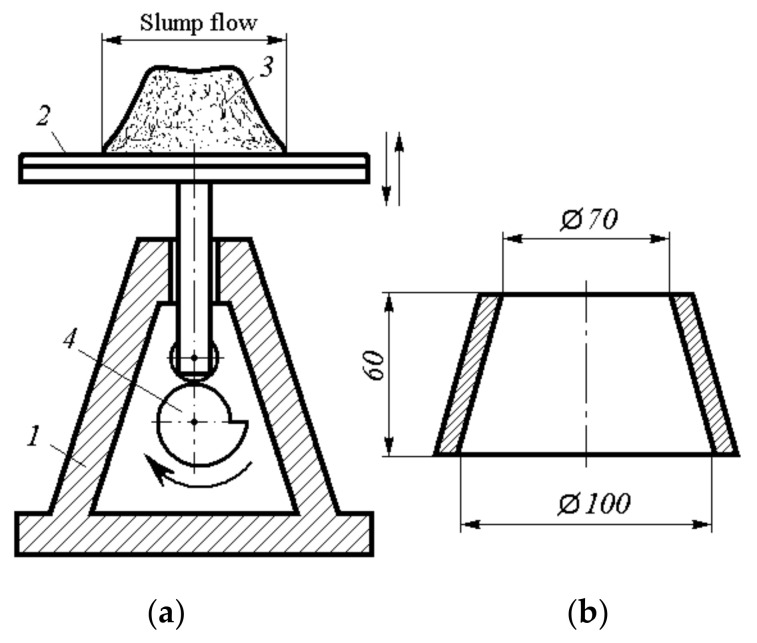
Flow table (**a**) and cone mould (**b**) for flow table test for mortar: 1 is base, 2 is flow table, 3 is cone from mortar, 4 is tappet.

First, the mortar was tested on specimens with dimensions of 40 × 40 × 160 mm for bending and compression, according to Russian State Standard GOST 30744-2001 “Cements. Methods of testing with using polyfraction standard sand” [31], with bending deformation measured according to a simplified scheme (series A and B). This made it possible to preliminarily evaluate the additive effect and range of possible dosages by the deflection value at three-point bending. Series A did not consider the water content of the MFC slurry, so the actual W/C increased with increasing Pro-Flowstab additive content, and the equal workability of the blends was controlled by SP addition. The mixture flowability was controlled by the cone spread on a flow table (see Figure 1a). The cone shape (see Figure 1b) was installed on the flow table and filled with mortar in two steps. Each layer was compacted with ten tamping rod presses. After removing the mold, 30 shakes were made and the cone spread of the mixture was measured in two mutually perpendicular directions. In test series B, the actual W/C was the same. 

**Figure 2:**
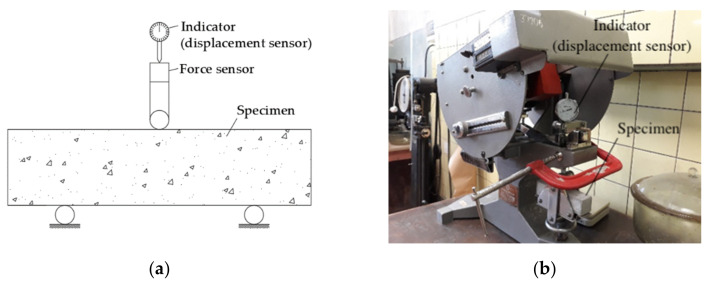
Setup for determining the strength and deflection in bending. (**a**) Testing scheme; (**b**) external view of the installation.

To preliminarily assess the effect of the MFC additive on the deformative properties of the material, the deflection of bending specimens with dimensions of 40 × 40 × 160 mm was determined according to the scheme shown in Figure 2 according to Russian State Standard GOST 30744-2001 [31].

We understand that this test scheme is not entirely correct for determining the modulus of deformation in bending, since the indicator readouts take into account the deflection of the beam and the deformation of the crushing of the material under the supports. However, this general deformation characterizes the material’s stiffness and allows the comparison of specimens in their deformation capacity.

The specimens were subjected to stepwise loading, holding at each step for 4–5 min, after which the readout was taken on a dial indicator with a division value of 1 μm. The loading was stopped at each stage when a certain equal stress level was reached. Three samples of each composition were tested. 

**Figure 3:**
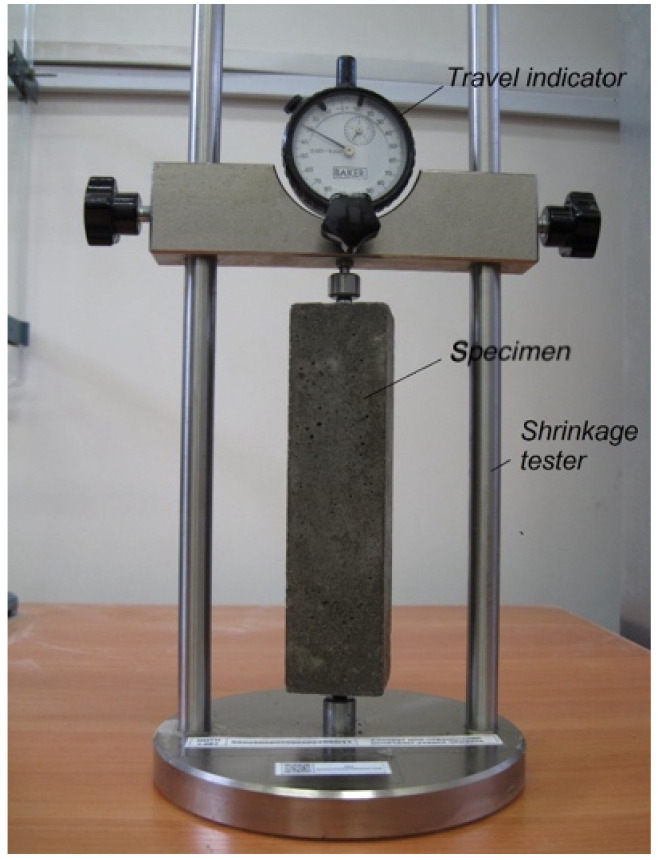
Device for measuring shrinkage of concrete specimens.

From mortar mixtures of series A, three specimens (with dimensions of 40 × 40 × 160 mm) of each composition were made in special shapes, making it possible to lay metal inserts along the ends of the specimens, serving as benchmarks for measuring shrinkage. Specimens in molds were stored in a chamber for standard hardening at a temperature of (20 ± 2 °C) and relative humidity (95 ± 5%). The specimens were removed from the mold one day after fabrication. After that, the measurement of the specimen length was started using a device equipped with a mercer clock gauge with a scale division of 1 μm (see Figure 3). During the shrinkage test, the specimens were kept in a climatic chamber at a temperature of (20 ± 2 °C) and relative humidity (60 ± 5%). Simultaneously, with shrinkage readings, the specimens were weighed, and the loss of water to evaporation was determined.

#### 2.2.2. Concrete Testing

The concrete tests were designated as series C. To determine the effect of Pro-Flowstab additive on the properties of concrete, 4 compositions were prepared that differ in the content of the MFC additive, which was 0; 1.0; 1.5; and 2% by weight of cement. The following parameters characterized concrete compositions: cement consumption C = 465 kg/m^3^; water-cement ratio W/C = 0.49, considering the water content in the MFC suspension; the proportion of sand in the mass of aggregates r = 0.40. The cone slump was selected by adjusting the dosage of the superplasticizer MC-TechniFlow 178 to maintain the uniformity of the mixtures. The dry matter content of the MFC suspension from the concrete volume was, respectively, 0.016; 0.024; and 0.032%.

**Figure 4:**
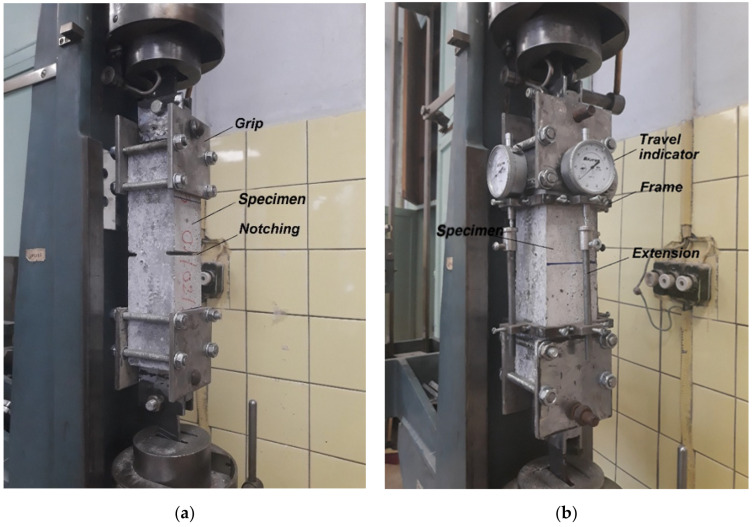
Testing Schemes. (**a**) For determination of tensile strength; (**b**) for determination of elastic modulus.

The ultimate compressive strength was determined on cube specimens 70 × 70 × 70 mm, according to Russian State Standard GOST 10180-2012 “Concretes. Methods for strength determination using reference specimens” [32]. Strength and modulus of deformation in tension was determined on prism specimens 70 × 70 × 280 mm, according to Russian State Standard GOST 24452-80 “Cements. Methods of testing with using polyfraction standard sand” [33]. Specimens were prepared from one batch of three specimens of each type for 2 test periods—7 and 28 days. A transverse notch with a depth of 10 mm was made on two opposite faces of the specimens (Figure 4a) to fix the rupture plane during tensile testing. The deformation was measured using a device with a mercer clock gauge with a scale division of 1 μm located on all four sides of the specimen (Figure 4b).

The tensile elastic modulus of concrete was determined following the Russian Standard GOST 24452-80 [34], for determining the modulus of elasticity in compression. Loading was performed in steps up to a load of 30–40% of the breaking load. At each stage, the specimens were kept under constant load for 4–5 min. When calculating the modulus of elasticity, only the deformation that occured with an increase in the load was considered. The creep areas during holding at the loading steps were discarded. Load shedding was performed continuously from the maximum value to the conditional zero. Deformation was measured with dial indicators located on all four sides of the specimen (Figure 4b).

The heat release of concrete Q was determined according to EN 196-9: 2010 [35], by the semi-adiabatic (thermos) method at an initial concrete temperature of 20 °C, and by calculation was reduced to an isothermal hardening regime at a temperature of 20 °C using the reduced time hypothesis [1], according to which at moments of equal heat release at Q_1_ = Q_2_, the ratio of the heat release rates, as well as the corresponding times τ_2_ and τ_1_, remains constant throughout the entire process:

(1) $$\frac{{\left(\partial Q/\partial \tau \right)}_{1}}{{\left(\partial Q/\partial \tau \right)}_{2}}=\frac{\tau_{1}}{\tau_{2}}=f_{t}=\mathrm{const}$$

The temperature function f_t_ was calculated by the formula:

(2) $$f_{t}=2^{\frac{t_{1}-t_{2}}{\varepsilon }}$$

where ε is the characteristic temperature difference if t_1_ − t_2_ = ε, then f_t_ = 2, that is, when the temperature rises by ε degrees, the heat release rate doubles. Two twin specimens of each concrete composition were tested. The readings of the temperature sensors were recorded by the Terem-4 multichannel meter recorder every 30 min.

The rules for holding the specimens and the test periods were adopted according to Russian State Standard GOST “Concretes. Methods for strength determination using reference specimens” 10180-2012 [32]. The samples were removed from molds one day after casting. Subsequently, the specimens were stored at a temperature of 20 ± 2 °C and relative humidity of at least 96%. After 28 days, the specimens were removed from the standard hardening chamber and dried to a constant weight, to exclude the influence of moisture.

## 3. Results and Discussion

### 3.1. Determination of Fexural and Compressive Strength of Cement-Sand Prism Specimens

The compositions of mortar mixtures are divided into two series, A and B (see Table 1). All compositions are characterized by a cement/sand ratio of 1:3. Pro-Flowstab additive was used as a ready-to-use slurry supplied by the manufacturer. The mobility of the mortar mixture was controlled by the addition of a superplasticizer. The difference between the series lies in a different approach to the purpose of the water-cement ratio. In series A, the mixing water consumption was taken without considering the water content in the MFC slurry, so that the actual W/C increased with increasing additive content. In series B, the mixing water consumption was taken considering the water content in the MFC suspension so that the actual W/C was 0.40. 

**Figure 5:**
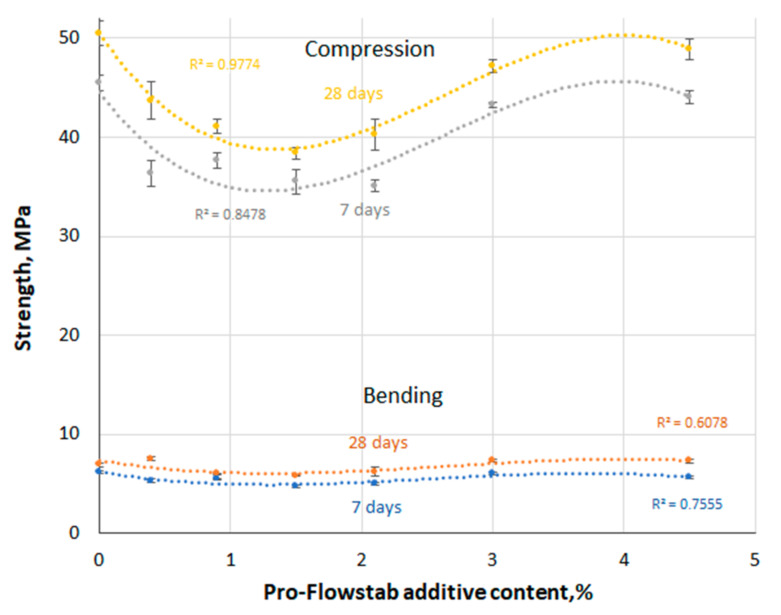
Results of determining the strength of series A specimens in bending and compression.

The results of testing cement specimens with dimensions of 40 × 40 × 160 mm are given in Table 2 and Figure 5.

Table 1 and Table 2 show that the addition of Pro-Flowstab requires a reduction in the amount of superplasticizer to obtain an equal mobility of the mixture. Hence, it follows that this additive increases the workability of the mixture, due to the additional amount of water contained in the suspension. At dosages of 0%, 0.9%, and 1.5%, the strength increased as a result of the inclusion of water in the additive suspension. At 3% addition, a decrease in strength occurred. However, it should be noted that the mobility of the mixture was regulated by the polycarboxylate.

From Table 2, it can be seen that, in general, the reference composition that does not contain the Pro-Flowstab additive remains the highest strength, both in bending and compression if some compositions’ flexural strength at the age of 28 days is not taken into account.

Tests show that strength does not change uniformly with increasing Pro-Flowstab additive content. However, in series A, there is a certain tendency in the change in strength, approximating a third degree polynomial (Figure 5). This tendency is observed in both early and late ages, both in flexion and compression. In this case, a wavy nature of the dependence of strength on the additive content is visible. With an increase in the amount of additive, the strength first decreases, reaching a minimum value at 2% additive content, after which it begins to increase, almost reaching the reference strength, and again begins to decrease. At the age of 28 days, with the extreme values of the additive content (0.4 and 4.5% of the cement weight), the flexural strength of the specimens exceeded the ultimate strength of the reference composition. The strength value decreases in the left section of the curves in Figure 5 could be explained by the increases in the water-cement ratio, due to additional water in the suspension. However, this phenomenon is not proper for the right section of the curves, since the strength increases with an increase in the actual W/C value.

The presence of extrema of the function usually indicates a change in the mechanism of influence, resulting from an oppositely acting competing factor, which in this case could be the reinforcing effect of the additive. However, testing the hypothesis by Fisher’s criterion did not confirm the adequacy of the model. In addition, an experimental verification was carried out by testing specimens with the same actual W/C, including the amount of water in the MFC dispersion (series B). At the same time, a decrease in the ultimate strength of the specimens in compression with an increase in the amount of additive was observed. Still, in the case of bending, a U-shaped curve was also observed, as in series A. Below, in experiments with concrete (series C), it is shown that the compressive strength clearly decreases with an increase in the additive content, but the flexural strength here also has a characteristic minimum at an intermediate dosage of MFC. It can also be assumed that at low dosages of the additive, the decrease in strength is caused by the negative effect of fatty acids contained in the membranes of parenchymal cells of microcellulose, and the insufficient reinforcing effect of the additive fibers. With an increase in dosage, the reinforcing effect is enhanced and surpasses the effect of organic compounds.

### 3.2. Determination of the Deflection in Bending of Cement-Sand Specimen Prisms

To preliminarily assess the effect of the MFC additive on the deformative properties of the material, the deflection of bending specimens with dimensions of 40 × 40 × 160 mm was determined, according to the scheme shown in Figure 2. When plotting the dependence of the bending stress σ on the conditional deflection f on the graphs, an average that was close to the linear portion of the curve was selected. The extreme points were discarded.

**Figure 6:**
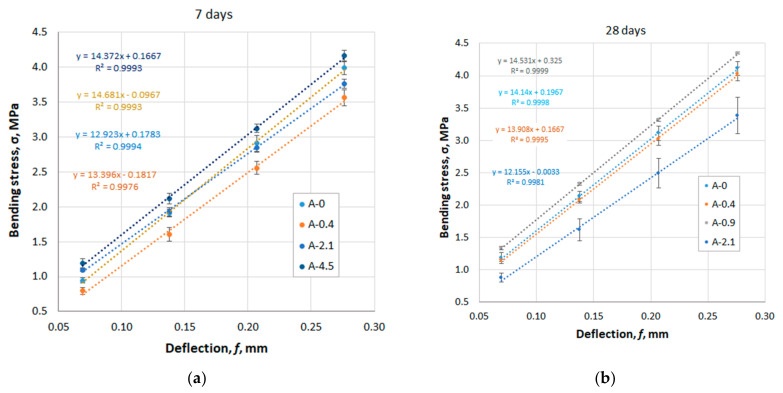
The results of measuring the deflection of specimen’s series A depending on the applied stress: (**a**) 7 days; (**b**) 28 days.

**Figure 7:**
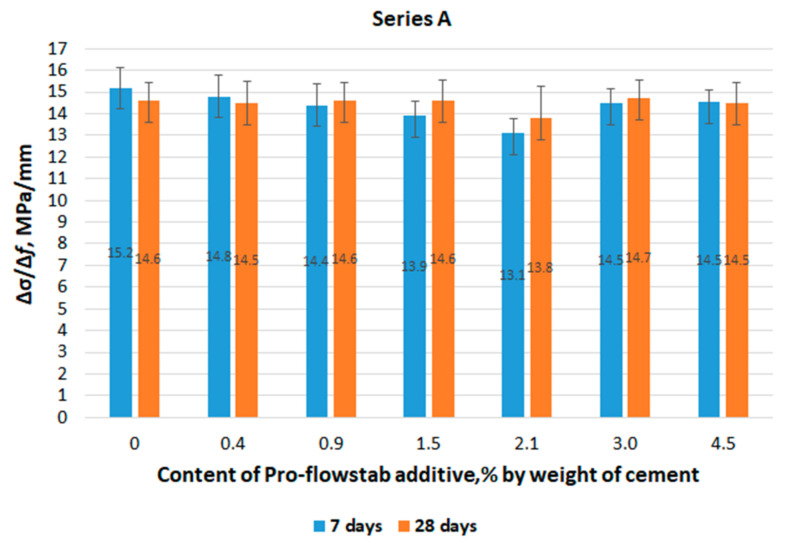
The value of the ratio ∆σ/∆f in the bending test of series A specimens, depending on the age of the specimens and the Pro-Flowstab additive contents.

The testing results of specimen series A are shown in Figure 6a,b and Figure 7.

Figure 6 shows an excellent linear correlation between the stress σ and the conditional deflection *f* with the coefficient of determination in the range R^2^ = 0.9905–0.9992. All curves lie in close proximity to each other and have close slope values, which indicates an insignificant discrepancy between the deformation properties of the compositions containing the Pro-Flowstab additive.

The angular coefficient values ∆σ/∆f depending on the content of the Pro-Flowstab additive and the age of the series A specimens are shown in Figure 7.

The ∆σ/∆f values of the specimens for 28 days of hardening are lower than those for 7 days, with the Pro-Flowstab additive content of 0 and 0.4% (see Figure 7), which does not correspond to other cases of the additive content. We assume this is due to the experimental error caused by measuring the deflection between the grips of the test machine, and not between the points of the specimen axis. This experiment is a preliminary comparative assessment of the effect of the additive on the deformative properties of the material. Nevertheless, regardless of the hardening period, the results show a tendency to a wave-like change in properties, similar to that observed in Figure 2 for strength. This confirms the above conclusion about the competing effect of W/C and the reinforcing effect of cellulose fibers.

The testing results of series B specimens are shown below.

**Figure 8:**
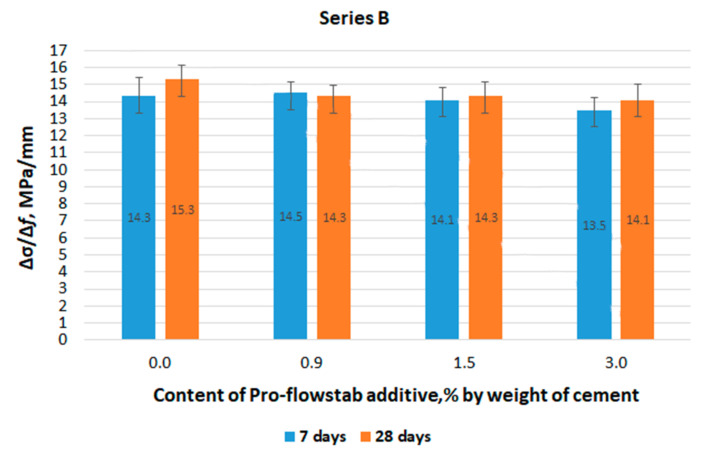
The value of the ratio ∆σ/∆*f* in the bending test of series B specimens, depending on the age of the specimens and the Pro-Flowstab additive contents.

The angular coefficient values ∆σ/∆f depending on the content of the Pro-Flowstab additive and the age of the series B specimens are shown in Figure 8.

In contrast to the specimens of series A, there is a synchronous decrease in the angular coefficient, and, consequently, in the deformation modulus with an increase in the Pro-Flowstab content. To assess the effect of age and compare the data for series A and B, Table 3 shows the averaged values of the slope ∆σ/∆*f* over series and ages.

According to Table 3, it can be concluded that:

1. Taking into account that the water contained in the additive in the W/C value had practically no effect on the test results;
2. The deformation capacity decreases insignificantly with the age of the specimens (at the same load ∆σ = const with an increase in ∆σ/∆f, the deformation ∆f decreases). This occurs as a result of an increase in the stiffness of the internal bonds.

### 3.3. Determination of Strength and Elastic Modulus of Concrete Specimens

#### 3.3.1. Strength

The test results of specimens for strength in compression and tensile are shown in Table 4.

As shown in Table 4, MFC additive reduces the strength of concrete in all types of tests (compression, tension, and bending). The higher the dosage of the additive, the more the strength decreases. The decrease in strength and retardation of hardening is explained by the hydrophilic nature of cellulose and the presence of organic compounds [36]. This circumstance is possibly related to the lack of special processing of cellulose fibers, since the quality of cement-fiber composites depends on the type of chemical treatment and the type of wood fiber [35].

#### 3.3.2. Modulus of Elasticity

**Figure 9:**
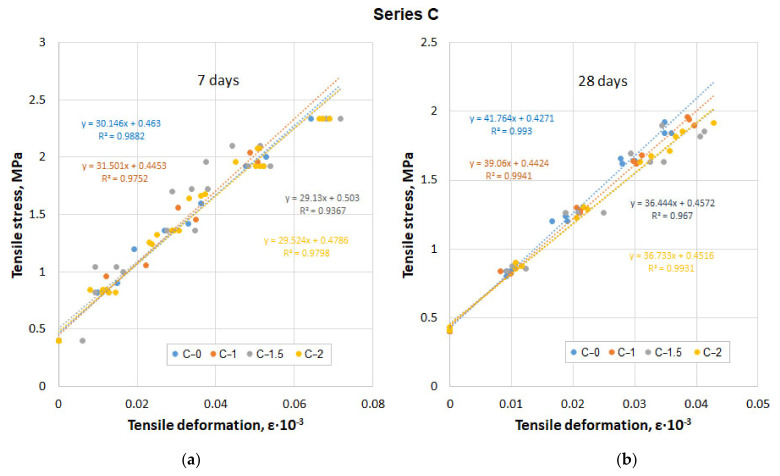
Results of measuring tensile deformation of series C specimens depending on the applied stress (**a**) 7 days; (**b**) 28 days.

The results of determining the tensile modulus of concrete are shown in Figure 9a,b.

**Figure 10:**
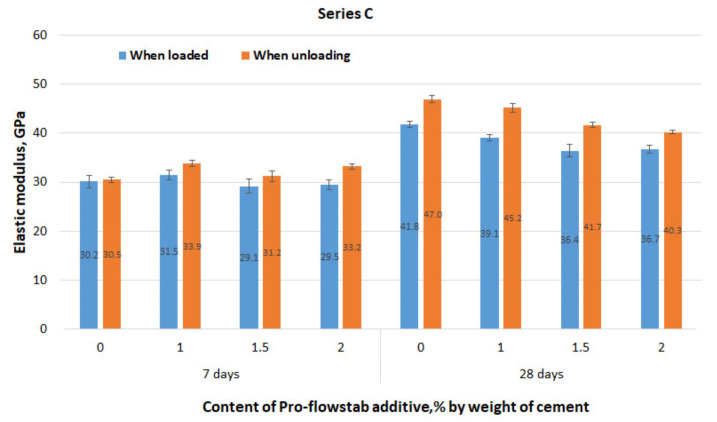
Values of tensile elastic modulus depending on the content of MFC additive.

The results of measuring the tensile strain of specimen series C, depending on the applied stress, are approximated by a linear dependence with the coefficient of determination R^2^ = 0.937−0.994, which is quite satisfactory. The graphs in Figure 9 reflect the results obtained with increasing tensile load. Similar graphs are not shown when the load is reduced. However, the final result in the form of the elastic modulus during unloading is demonstrated in Figure 10 compared to the elastic modulus under loading.

As shown in Figure 10, the modulus of elasticity calculated when the load falls, in all cases, exceeds the value of the modulus obtained under loading. This exceedance is explained by the fact that only reversible elastic deformation occurs during specimen unloading. At the concrete’s age of 7 days, there is a slight increase in the modulus of elasticity compared to the reference composition with a Pro-Flowstab content of 1%. However, with a further increase in this additive’s dosage, the elastic modulus decreases and becomes below the reference. In a period of 28 days, there was a more obvious tendency towards the decrease in elastic modulus with an increase in the dosage of Pro-Flowstab.

### 3.4. Determining the Effect of Pro-Flowstab Additive on the Heat Release of Concrete

**Figure 11:**
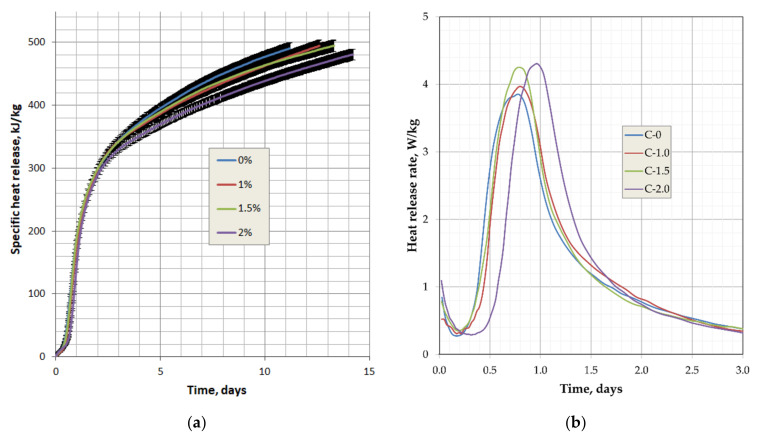
Influence of Pro-Flowstab additive: (**a**) on the integral value; (**b**) rate of the specific heat flow of cement in concrete.

The effect of Pro-Flowstab on the cumulative heat release per 1 kg of cement q = Q/C is shown in Figure 11a. Figure 11b shows the curves of the heat flow dq/dτ.

Figure 11 shows the curves of the specific heat release and the rate of exotherm of cement in concrete with an additive content of 1%, 1.5%, and 2%. The curves in Figure 11a have the segments representing the standard deviation of the average heat release obtained in three identical experiments. Since the experimental points were taken every 0.5 h, these segments merged into a solid dark area. These areas intersect for curves C-0 and C-1, from which it follows that a small amount of up to 1% additive does not affect the heat release of concrete, or it does, but only slightly. If we do not take into account the results for 1% additive content and compare the three remaining curves, then the following conclusions can be drawn.

The total amount of heat (Figure 11a) released by cement during hydration decreases with the introduction of Pro-Flowstab additives. An increase in the additive dosage leads to an increasingly noticeable decrease in the exothermic effect, starting from more than 2 days. The Pro-Flowstab additive had the most significant impact on the heat release of concrete at a content of 2% (composition C-2.0). With an additive content of 1 and 1.5% (compositions C-1.0 and C-1.5), the heat release of concrete is practically the same and differs little from the exotherm of the reference. When comparing the curves of the rate of heat release dq/dτ (Figure 11b), it can be seen that with an additive content of up to 1%, the rate of heat release by concrete is practically the same as that of the reference composition. The dq/dτ value for composition C-1.5 differs from the previous ones only in the peak height. Composition C-2.0 is characterized by a shift of the dq/dτ curve towards later dates. In this case, a more extended induction period takes place (retardation of setting and a higher value of the peak of the heat release rate). Thus, increasing the additive dosage causes a decrease in the exotherm rate at an early age up to 0.8−1.0 day. This is consistent with the results of determining the concrete strength (see Table 4), because both strength and heat release depend on the rate of cement hydration.

### 3.5. Determining the Effect of Pro-Flowstab Additive on Concrete Shrinkage

**Figure 12:**
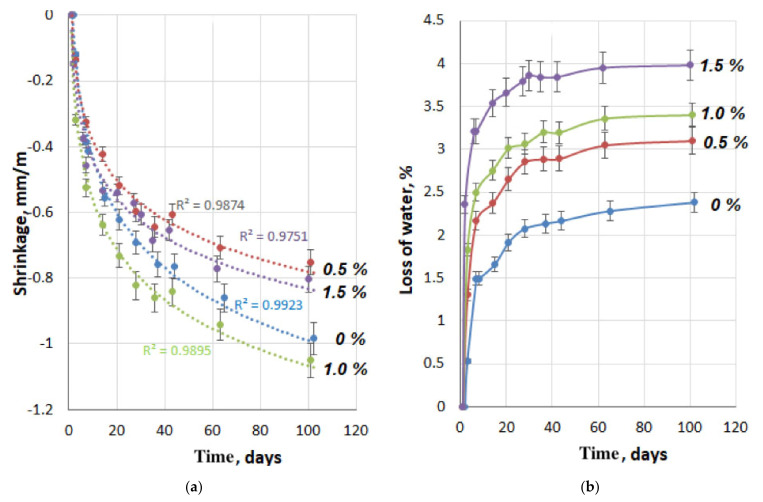
Influence of Pro-Flowstab additive: (**a**) on-air shrinkage deformation; (**b**) water loss for series B specimens.

**Figure 13:**
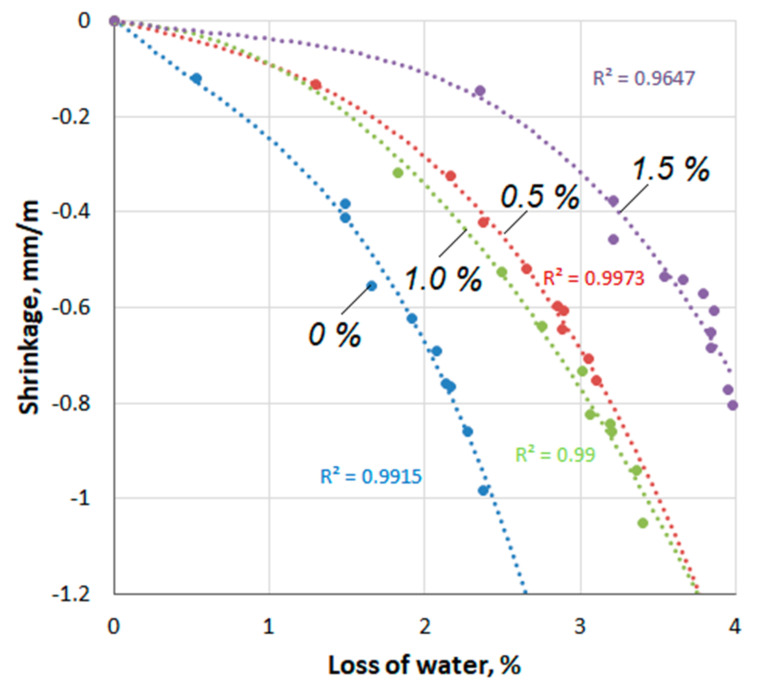
Dependence of shrinkage deformation on the amount of evaporated water.

The results of testing the compositions for linear shrinkage are shown in Figure 12a,b and Figure 13. The dependence of concrete shrinkage on the hurdening time (Figure 12a) is approximated by a logarithmic function with a good approximation (R2 = 0.975–0.992). As can be seen from Figure 12a, the addition of Pro-Flowstab additive in small (0.5%) and large (1.5%) amounts reduces shrinkage in comparison with the reference, and at its intermediate content (1%), the shrinkage is higher than in the reference specimens. A similar non-uniform effect of the additive was also observed in determining the strength and deformation capacity of cement-sand specimens of series A. As shown in Figure 12b, the addition of Pro-Flowstab accelerates the evaporation of water from the solution. In this case, the rate of evaporation increases with an increase in the content of additives. At the same time, there is evidence [6] that the inclusion of cellulose in parenchymal cells increases the control of fluid loss in circulating drilling fluids. A saturation of 80% is reached in just 2.5 min [36]. Figure 13 shows the dependence of the shrinkage deformation on the amount of evaporated water. This dependence correlates well with the third degree polynomial (R2 = 0.975–0.992). With the same water loss by the specimens, shrinkage decreases with an increase in the Pro-Flowstab content. On the other hand, the specimens with the additive must lose more water to achieve the same shrinkage value. It is possible that the additional amount of water contained in the MFC suspension affected this. If so, the shrinkage reducing the effect of the additive is even more significant.

## 4. Discussion

Testing of cement-sand specimens of prisms showed that in most cases the control composition, which did not contain the Pro-Flowstab additive, had the greatest strength, both in bending and in compression. With an increase in the amount of additive, starting from 0.4% by weight of cement to 0.9−1.5%, a decrease in strength was observed. With a further increase in the dosage to 3−4.5%, the strength increased but remained below the strength of the reference. In the case of concrete tests, compressive strength decreases more significantly than tensile strength. So, at a dosage of Pro-Flowstab 1, 1.5, and 2%, and the age of the specimens being 28 days, the compressive strength decreased, respectively, by 3.1, 11.7, and 22.3%, and tensile strength by 6.4, 7.4, and 18.9%, compared to the strength of the reference. These results are consistent with several sources that indicated a slowdown in hydration and a decrease in the strength of cement-based mixtures in the presence of MFC [10,11,12,13,14,15,16]. Responsibility for this lies with the chemical components and soluble sugars contained in plant fibers. It is also known that MFC improves strength later. Increasing the Pro-Flowstab additive dosage increases the concrete mixture’s workability, which is also consistent with the literature data.

There was a good linear correlation between the stress σ and the conditional deflection f with the coefficient of determination in the range R^2^ = 0.9905−0.9992. All compositions have close values of the angular coefficients σ/f, which indicates an insignificant discrepancy in the deformation properties of mortar, containing and not containing the Pro-Flowstab additive. In the case of concrete, the additive affected the modulus of elasticity differently at the age of the specimens, 7 and 28 days. The concrete showed a slight increase in the modulus of elasticity at 7 days of age and a decrease at 28 days.

The integral heat released by cement during hydration in concrete is reduced in the presence of Pro-Flowstab, starting from a period of about 2 days. At a low additive content of 1 and 1.5%, the heat release of concrete slightly differs from the exotherm of the reference. The most significant decrease in heat release to concrete that was aged 11 days was observed by adding Pro-Flowstab at a content of 2%. For the composition with a 2% content of the additive, the curve of the heat release rate dq/dτ shifted towards later periods, i.e., an increase in the duration of the induction period. The increase confirms the results of other authors on the retardation of cement hydration in the early stages of hardening. At the same time, a higher hydration peak value may mean that the Pro-Flowstab additive enhances hydration.

With an additive content of 0.5%, the shrinkage of the specimens by the age of 100 days decreased in comparison with the reference by about 20%. At the same time, it is interesting that with a lower shrinkage, by this time this concrete had lost about 30% more of the amount to water evaporation than the reference composition. With an increase in the dosage of the additive, the amount of evaporated moisture increasesd. So, with an additive content of 1.5%, the difference in water loss was about 67%. This circumstance is probably explained by the fact that cellulose fibers restrain the convergence processes of tobermorite gel particles and their compaction. On the other hand, the gaps between the gel particles, while remaining enlarged, retain moisture with less energy and increase its evaporation rate.

A feature of the tested additive is its non-monotonic wave-like effect on the properties of concrete, depending on the dosage. Perhaps this is a consequence of increased acidity (pH = 4), due to the presence of fatty acids. These fatty acids are found mainly in the parenchymal cells of plants, from the primary membrane of which this type of microcellulose is obtained. In any case, this is a matter for further research and improvement of the production technology.

## 5. Conclusions

Pro-Flowstab additive appeared on the construction market relatively recently, and is poorly studied. In this work, experimental studies on the effect of the Pro-Flowstab additive on the strength, deformability, heat release, and air shrinkage of cement composites are carried out since these properties determine the crack resistance of concrete.

1. For mortar with an increase in the content of additive Pro-Flowstab, a decrease in strength was observed, both in bending and in compression. Similar results were obtained in the case of concrete tests. Compression, tensile, and flexural tests of concrete showed a decrease in strength in the presence of Pro-Flowstab additive. Compressive strength decreases more significantly than tensile strength.
2. The addition of Pro-Flowstab to cementitious composites requires reducing either the amount of superplasticizer or the water/cement ratio to obtain equal flow. Adjustment of the workability of the mixture in one and the other way did not show significant differences in strength and deformation properties.
3. Adding Pro-Flowstab additive does not significantly affect the deformation properties of mortar. For concrete, the presence of Pro-Flowstab admixture significantly affects the modulus of elasticity. An increase in the dosage of the Pro-Flowstab additive by 0.5% of the cement mass reduces the elastic modulus of concrete by 1.75 GPa. From the point of view of the crack resistance of concrete, this circumstance is positive, provided that the strength remains unchanged.
4. Pro-Flowstab additive reduces the integral heat released by cement during hydration, and, at the same time, delays this process. However, the higher hydration peak value may mean the general tendency is that the Pro-Flowstab enhances hydration.
5. Pro-Flowstab additive has a positive effect on the air shrinkage of concrete. The higher the content of Pro-Flowstab, the higher the concrete shrinkage, with the same amount of lost water. On the other hand, the specimens with the additive must lose more water to achieve the same shrinkage value.
6. Thus, the Pro-Flowstab additive, providing concrete with lower elastic modulus values, heat release, and shrinkage, can be recommended for use in concretes with increased crack resistance during the hardening period. The recommended additive content is 0.5% by weight of cement. There is an insignificant loss in strength with this content but a significant decrease in concrete shrinkage with moderate heat release. At the specified dosage, it is possible to provide the class of concrete in terms of compressive strength C35/45 following Russian State Standard GOST 57345-2016 /EN 206-1:2013 “Concrete. General specifications”.

## Figures and Tables

**Table 1:** Consumption of materials for the investigated compositions.

Designation	MFC Content		Consumption of Materials, kg/m^3^					Slump Flow, mm
	In % of Suspension from Cement Consumption	In % Dry Matter by Volume	C	W	S	SP	MFC	
A-0	0	0	543	217	1629	5.4	0.0	129
A-0.4	0.4	0.005	542	217	1624	5.4	2.4	137
A-0.9	0.9	0.011	541	216	1619	5.4	4.8	125
A-1.5	1.5	0.018	540	216	1612	5.4	8.1	133
A-2.1	2.1	0.025	539	216	1606	4.8	11.4	126
A-3.0	3.0	0.035	537	215	1596	5.4	16.1	126
A-4.5	4.5	0.053	537	215	1576	4.5	24.2	134
B-0	0	0	542	217	1627	6.3	0	120
B-0.9	0.9	0.011	544	213	1627	5.0	4.8	120
B-1.5	1.5	0.018	545	210	1625	5.2	8.2	118
B-3.0	3.0	0.036	546	202	1621	5.5	16.4	122

**Table 2:** Results of bending and compression testing of series B prism specimens 40 × 40 × 160 mm.

Designation	Slump Flow, mm	Flexural Strength, MPa, Age, Day		Standard Deviation		Compressive Strength, MPa, Age, Day		Standard Deviation	
		7	28	7	28	7	28	7	28
B-0	120	5.89	6.76	0.292	0.115	46.4	52.5	0.99	1.14
B-0.9	120	5.79	7.05	0.232	0.177	46.5	51.4	1.72	1.87
B-1.5	118	6.42	5.96	0.202	0.072	43.8	45.3	1.56	0.65
B-3.0	122	5.54	6.16	0.176	0.29	36.8	44.2	0.95	2.24

**Table 3:** Averaged over series and age values of the slope ∆σ/∆*f*.

Specimen Age, Days	The Values of the Angular Coefficient Δσ/Δ*f*, MPa/mm	
	Serie A	Serie B
7	14.3	14.1
28	14.5	14.5

**Table 4:** Test results of specimens for strength in compression and tensile.

Specimen Age, Days	Designation	MFC Content, % by Weight of Cement	Ultimate Strength, MPa			
			Tensile	Standard Deviation	Compression	Standard Deviation
7	C-0	0	3.81	0.36	46.08	1.41
	C-1	1	3.94	0.42	44.61	0.78
	C-1.5	1.5	3.88	0.21	41.67	1.39
	C-2	2	3.67	0.38	39.44	1.10
28	C-0	0	4.87	0.37	57.80	1.69
	C-1	1	4.56	0.12	56.00	1.68
	C-1.5	1.5	4.51	0.49	51.05	1.47
	C-2	2	3.95	0.17	44.89	1.65
